# 1-(4-Chloro­phen­yl)-3-(2-nitro­phenyl)propane-1,2-dione

**DOI:** 10.1107/S1600536808030559

**Published:** 2008-09-27

**Authors:** Lin Huang, Shuqin Li, Huisheng Li

**Affiliations:** a Department of Chemistry and Biology, Xiangfan University, Xiangfan 441053, People’s Republic of China

## Abstract

The title compound, C_15_H_10_ClNO_4_, belongs to the class of 1,2-diketones, which have important applications in both synthetic organic chemistry and supra­molecular chemistry. A dihedral angle of 9.03 (1)° is found between the mean planes of the two benzene rings. C—H⋯O inter­actions help to stabilize the crystal structure.

## Related literature

For the synthesis of the title compound, see: Barnes & Gist (1950 ▶). For applications of the title compound, see: Saalfrank *et al.* (1988 ▶); Schobert (1988 ▶); van Leusen & van Leusen (1977 ▶).
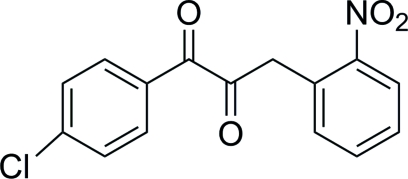


## Experimental

### 

#### Crystal data

C_15_H_10_ClNO_4_
*M* *_r_* = 303.69
Monoclinic, 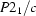

*a* = 7.9995 (3) Å
*b* = 6.3730 (2) Å
*c* = 26.7750 (5) Å
β = 91.340 (2)°
*V* = 1364.64 (7) Å^3^
*Z* = 4
Mo *K*α radiation
μ = 0.29 mm^−1^
*T* = 298 (2) K
0.20 × 0.10 × 0.10 mm

#### Data collection

Bruker SMART 4K CCD area-detector diffractometer
Absorption correction: multi-scan (*SADABS*; Sheldrick, 1997 ▶) *T*_min_ = 0.943, *T*_max_ = 0.971
8762 measured reflections
2408 independent reflections
1872 reflections with *I* > 2σ(*I*)
*R*_int_ = 0.027

#### Refinement

*R*[*F* ^2^ > 2σ(*F* ^2^)] = 0.047
*wR*(*F* ^2^) = 0.133
*S* = 1.07
2408 reflections
190 parameters
H-atom parameters constrained
Δρ_max_ = 0.38 e Å^−3^
Δρ_min_ = −0.28 e Å^−3^

### 

Data collection: *SMART* (Bruker, 2001 ▶); cell refinement: *SAINT* (Bruker, 1999 ▶); data reduction: *SAINT*; program(s) used to solve structure: *SHELXS97* (Sheldrick, 2008 ▶); program(s) used to refine structure: *SHELXL97* (Sheldrick, 2008 ▶); molecular graphics: *SHELXTL* (Sheldrick, 2008 ▶); software used to prepare material for publication: *SHELXTL*.

## Supplementary Material

Crystal structure: contains datablocks I, global. DOI: 10.1107/S1600536808030559/cs2095sup1.cif

Structure factors: contains datablocks I. DOI: 10.1107/S1600536808030559/cs2095Isup2.hkl

Additional supplementary materials:  crystallographic information; 3D view; checkCIF report

## Figures and Tables

**Table 1:** Hydrogen-bond geometry (Å, °)

*D*—H⋯*A*	*D*—H	H⋯*A*	*D*⋯*A*	*D*—H⋯*A*
C5—H5⋯O3^i^	0.93	2.53	3.279 (3)	138
C15—H15⋯O1	0.93	2.59	3.244 (3)	128

Symmetry code: (i) 
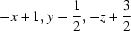
.

**Fig. 1.**
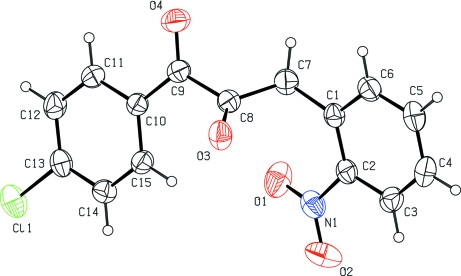
View of the title molecule showing the atom-labelling scheme. Displacement ellipsoids are drawn at the 30% probability level. H atoms are represented by spheres of arbitrary radius.

## supplementary crystallographic information

### Comment

1,2-Diketones are important intermediates in the preparation of heterocyclic compounds in synthetic chemistry (van Leusen & van Leusen, 1977; Saalfrank *et al.*, 1988). In addition, they can also be used as ligands in supramolecular chemistry (Schobert, 1988). The molecular structure of the title compound is shown in Fig. 1. The dihedral angle of the two benzene rings is 9.03 (1)°. Molecules are mainly connected by an intermolecular C—H···O interaction.

### Experimental

The title compound was synthesized as previously described by Barnes & Gist (1950). Colorless crystals suitable for X-ray data collection were obtained by slow evaporation of a 5:2 ratio CH_2_Cl_2_:MeOH solution at 293 K.

### Refinement

All H atoms were positioned geometrically (C—H = 0.93–0.97 Å) and refined using a riding model, with *U*_iso_(H) = 1.2 *U*_eq_(C) (1.5*U*_eq_(C) for methyl) of the parent atoms.

### Crystal data

**Table:**

C_15_H_10_ClNO_4_	*F*(000) = 624
*M**_r_* = 303.69	*D*_x_ = 1.478 Mg m^−^^3^
Monoclinic, *P*2_1_/*c*	Mo *K*α radiation, λ = 0.71073 Å
Hall symbol: -P 2ybc	Cell parameters from 2433 reflections
*a* = 7.9995 (3) Å	θ = 2.6–23.4°
*b* = 6.3730 (2) Å	µ = 0.30 mm^−^^1^
*c* = 26.7750 (5) Å	*T* = 298 K
β = 91.340 (2)°	Block, colourless
*V* = 1364.64 (7) Å^3^	0.20 × 0.10 × 0.10 mm
*Z* = 4	

### Data collection

**Table:**

Bruker SMART 4K CCD area-detector diffractometer	2408 independent reflections
Radiation source: fine-focus sealed tube	1872 reflections with *I* > 2σ(*I*)
graphite	*R*_int_ = 0.027
φ and ω scans	θ_max_ = 25.0°, θ_min_ = 2.6°
Absorption correction: multi-scan (SADABS; Sheldrick, 1997)	*h* = −9→9
*T*_min_ = 0.943, *T*_max_ = 0.971	*k* = −7→7
8762 measured reflections	*l* = −31→31

### Refinement

**Table:**

Refinement on *F*^2^	Primary atom site location: structure-invariant direct methods
Least-squares matrix: full	Secondary atom site location: difference Fourier map
*R*[*F*^2^ > 2σ(*F*^2^)] = 0.047	Hydrogen site location: inferred from neighbouring sites
*wR*(*F*^2^) = 0.133	H-atom parameters constrained
*S* = 1.07	*w* = 1/[σ^2^(*F*_o_^2^) + (0.0688*P*)^2^ + 0.2924*P*] where *P* = (*F*_o_^2^ + 2*F*_c_^2^)/3
2408 reflections	(Δ/σ)_max_ < 0.001
190 parameters	Δρ_max_ = 0.38 e Å^−^^3^
0 restraints	Δρ_min_ = −0.28 e Å^−^^3^

### Fractional atomic coordinates and isotropic or equivalent isotropic displacement parameters (Å^2^)

**Table:**

	*x*	*y*	*z*	*U*_iso_*/*U*_eq_	
C1	0.4590 (2)	0.5187 (3)	0.67527 (7)	0.0496 (5)	
C2	0.4028 (3)	0.7133 (3)	0.65828 (7)	0.0498 (5)	
C3	0.2727 (3)	0.8197 (4)	0.68005 (9)	0.0606 (6)	
H3	0.2382	0.9492	0.6676	0.073*	
C4	0.1952 (3)	0.7326 (5)	0.72019 (9)	0.0675 (7)	
H4	0.1075	0.8028	0.7351	0.081*	
C5	0.2471 (3)	0.5421 (4)	0.73831 (9)	0.0669 (7)	
H5	0.1947	0.4829	0.7656	0.080*	
C6	0.3771 (3)	0.4379 (4)	0.71616 (8)	0.0588 (6)	
H6	0.4111	0.3089	0.7291	0.071*	
C7	0.6000 (3)	0.3935 (4)	0.65391 (9)	0.0602 (6)	
H7A	0.6018	0.2561	0.6695	0.072*	
H7B	0.5777	0.3735	0.6185	0.072*	
C8	0.7691 (3)	0.4913 (3)	0.66072 (7)	0.0504 (5)	
C9	0.9136 (3)	0.3769 (4)	0.63586 (8)	0.0539 (5)	
C10	1.0144 (2)	0.4879 (3)	0.59876 (7)	0.0471 (5)	
C11	1.1423 (3)	0.3809 (4)	0.57538 (8)	0.0568 (6)	
H11	1.1665	0.2433	0.5846	0.068*	
C12	1.2338 (3)	0.4757 (4)	0.53865 (8)	0.0631 (6)	
H12	1.3183	0.4029	0.5228	0.076*	
C13	1.1976 (3)	0.6808 (4)	0.52582 (8)	0.0571 (6)	
C14	1.0729 (3)	0.7893 (4)	0.54892 (8)	0.0572 (6)	
H14	1.0505	0.9278	0.5401	0.069*	
C15	0.9814 (3)	0.6933 (3)	0.58507 (8)	0.0536 (5)	
H15	0.8965	0.7669	0.6005	0.064*	
Cl1	1.31213 (9)	0.80635 (13)	0.48046 (2)	0.0813 (3)	
N1	0.4783 (3)	0.8163 (4)	0.61492 (7)	0.0653 (6)	
O1	0.5769 (2)	0.7162 (4)	0.58971 (7)	0.0969 (7)	
O2	0.4318 (3)	0.9934 (3)	0.60410 (8)	0.0994 (7)	
O3	0.79710 (19)	0.6406 (3)	0.68656 (6)	0.0693 (5)	
O4	0.9394 (2)	0.1971 (3)	0.64872 (8)	0.0855 (6)	

### Atomic displacement parameters (Å^2^)

**Table:**

	*U*^11^	*U*^22^	*U*^33^	*U*^12^	*U*^13^	*U*^23^
C1	0.0428 (11)	0.0539 (13)	0.0520 (11)	−0.0072 (9)	−0.0024 (9)	−0.0057 (10)
C2	0.0441 (11)	0.0588 (14)	0.0463 (11)	−0.0084 (10)	−0.0063 (9)	−0.0001 (10)
C3	0.0509 (13)	0.0614 (15)	0.0687 (15)	0.0020 (11)	−0.0109 (11)	−0.0052 (11)
C4	0.0447 (13)	0.0881 (19)	0.0697 (15)	0.0040 (12)	0.0022 (11)	−0.0143 (14)
C5	0.0516 (14)	0.0881 (19)	0.0614 (14)	−0.0112 (13)	0.0082 (11)	0.0013 (13)
C6	0.0538 (13)	0.0602 (14)	0.0623 (13)	−0.0091 (11)	−0.0032 (10)	0.0065 (11)
C7	0.0574 (14)	0.0606 (14)	0.0627 (13)	−0.0031 (11)	0.0040 (10)	−0.0117 (11)
C8	0.0533 (13)	0.0521 (13)	0.0461 (11)	0.0014 (10)	0.0050 (9)	0.0032 (10)
C9	0.0544 (13)	0.0497 (13)	0.0579 (12)	0.0040 (10)	0.0038 (10)	0.0016 (10)
C10	0.0448 (11)	0.0478 (12)	0.0485 (11)	0.0002 (9)	−0.0008 (9)	−0.0050 (9)
C11	0.0542 (13)	0.0491 (13)	0.0676 (14)	0.0044 (10)	0.0066 (10)	−0.0056 (10)
C12	0.0537 (14)	0.0705 (17)	0.0655 (14)	0.0015 (12)	0.0141 (11)	−0.0154 (12)
C13	0.0509 (13)	0.0724 (17)	0.0479 (12)	−0.0099 (11)	0.0004 (10)	−0.0030 (10)
C14	0.0580 (13)	0.0548 (13)	0.0589 (13)	−0.0014 (11)	0.0016 (10)	0.0049 (10)
C15	0.0492 (12)	0.0549 (14)	0.0568 (12)	0.0055 (10)	0.0047 (9)	0.0001 (10)
Cl1	0.0776 (5)	0.1050 (6)	0.0619 (4)	−0.0200 (4)	0.0179 (3)	0.0070 (3)
N1	0.0593 (12)	0.0772 (16)	0.0588 (12)	−0.0135 (11)	−0.0082 (10)	0.0125 (11)
O1	0.0753 (13)	0.143 (2)	0.0732 (13)	0.0231 (13)	0.0198 (10)	0.0355 (12)
O2	0.140 (2)	0.0620 (13)	0.0966 (14)	−0.0183 (13)	0.0092 (13)	0.0166 (11)
O3	0.0546 (9)	0.0763 (11)	0.0774 (11)	−0.0077 (8)	0.0083 (8)	−0.0257 (9)
O4	0.0905 (14)	0.0608 (12)	0.1068 (14)	0.0187 (10)	0.0394 (11)	0.0232 (10)

### Geometric parameters (Å, °)

**Table:**

C1—C6	1.388 (3)	C8—C9	1.532 (3)
C1—C2	1.392 (3)	C9—O4	1.213 (3)
C1—C7	1.505 (3)	C9—C10	1.475 (3)
C2—C3	1.382 (3)	C10—C15	1.383 (3)
C2—N1	1.475 (3)	C10—C11	1.390 (3)
C3—C4	1.371 (3)	C11—C12	1.379 (3)
C3—H3	0.9300	C11—H11	0.9300
C4—C5	1.368 (4)	C12—C13	1.380 (3)
C4—H4	0.9300	C12—H12	0.9300
C5—C6	1.380 (3)	C13—C14	1.373 (3)
C5—H5	0.9300	C13—Cl1	1.734 (2)
C6—H6	0.9300	C14—C15	1.371 (3)
C7—C8	1.497 (3)	C14—H14	0.9300
C7—H7A	0.9700	C15—H15	0.9300
C7—H7B	0.9700	N1—O2	1.221 (3)
C8—O3	1.195 (3)	N1—O1	1.229 (3)
			
C6—C1—C2	115.7 (2)	C7—C8—C9	115.99 (19)
C6—C1—C7	118.3 (2)	O4—C9—C10	123.5 (2)
C2—C1—C7	126.0 (2)	O4—C9—C8	116.8 (2)
C3—C2—C1	122.7 (2)	C10—C9—C8	119.63 (19)
C3—C2—N1	116.1 (2)	C15—C10—C11	118.9 (2)
C1—C2—N1	121.2 (2)	C15—C10—C9	121.91 (19)
C4—C3—C2	119.4 (2)	C11—C10—C9	119.2 (2)
C4—C3—H3	120.3	C12—C11—C10	120.9 (2)
C2—C3—H3	120.3	C12—C11—H11	119.5
C5—C4—C3	119.9 (2)	C10—C11—H11	119.5
C5—C4—H4	120.1	C11—C12—C13	118.7 (2)
C3—C4—H4	120.1	C11—C12—H12	120.6
C4—C5—C6	120.0 (2)	C13—C12—H12	120.6
C4—C5—H5	120.0	C14—C13—C12	121.0 (2)
C6—C5—H5	120.0	C14—C13—Cl1	119.0 (2)
C5—C6—C1	122.3 (2)	C12—C13—Cl1	119.99 (18)
C5—C6—H6	118.8	C15—C14—C13	119.9 (2)
C1—C6—H6	118.8	C15—C14—H14	120.0
C8—C7—C1	114.60 (19)	C13—C14—H14	120.0
C8—C7—H7A	108.6	C14—C15—C10	120.5 (2)
C1—C7—H7A	108.6	C14—C15—H15	119.7
C8—C7—H7B	108.6	C10—C15—H15	119.7
C1—C7—H7B	108.6	O2—N1—O1	123.0 (2)
H7A—C7—H7B	107.6	O2—N1—C2	118.0 (2)
O3—C8—C7	123.96 (19)	O1—N1—C2	118.8 (2)
O3—C8—C9	119.84 (19)		
			
C6—C1—C2—C3	−0.7 (3)	O4—C9—C10—C15	−179.9 (2)
C7—C1—C2—C3	−179.6 (2)	C8—C9—C10—C15	−0.7 (3)
C6—C1—C2—N1	−179.61 (18)	O4—C9—C10—C11	2.6 (3)
C7—C1—C2—N1	1.4 (3)	C8—C9—C10—C11	−178.20 (19)
C1—C2—C3—C4	0.3 (3)	C15—C10—C11—C12	−0.9 (3)
N1—C2—C3—C4	179.30 (19)	C9—C10—C11—C12	176.7 (2)
C2—C3—C4—C5	0.1 (3)	C10—C11—C12—C13	0.8 (3)
C3—C4—C5—C6	−0.1 (4)	C11—C12—C13—C14	−0.1 (3)
C4—C5—C6—C1	−0.3 (4)	C11—C12—C13—Cl1	179.04 (17)
C2—C1—C6—C5	0.7 (3)	C12—C13—C14—C15	−0.5 (3)
C7—C1—C6—C5	179.7 (2)	Cl1—C13—C14—C15	−179.67 (17)
C6—C1—C7—C8	−112.2 (2)	C13—C14—C15—C10	0.4 (3)
C2—C1—C7—C8	66.7 (3)	C11—C10—C15—C14	0.2 (3)
C1—C7—C8—O3	9.9 (3)	C9—C10—C15—C14	−177.2 (2)
C1—C7—C8—C9	−175.36 (19)	C3—C2—N1—O2	6.2 (3)
O3—C8—C9—O4	115.7 (3)	C1—C2—N1—O2	−174.8 (2)
C7—C8—C9—O4	−59.3 (3)	C3—C2—N1—O1	−169.5 (2)
O3—C8—C9—C10	−63.5 (3)	C1—C2—N1—O1	9.5 (3)
C7—C8—C9—C10	121.5 (2)		

### Hydrogen-bond geometry (Å, °)

**Table:**

*D*—H···*A*	*D*—H	H···*A*	*D*···*A*	*D*—H···*A*
C5—H5···O3^i^	0.93	2.53	3.279 (3)	138
C15—H15···O1	0.93	2.59	3.244 (3)	128

Symmetry codes: (i) −*x*+1, *y*−1/2, −*z*+3/2.
